# Comparison of Fatty Acid Contents and MMP-1 Inhibitory Effects of the Two Antarctic Fish, *Notothenia rossii* and *Champsocephalus gunnari*

**DOI:** 10.3390/molecules27144554

**Published:** 2022-07-17

**Authors:** Seulah Lee, Man Hyung Koo, Dong-Won Han, Il-Chan Kim, Jun Hyuck Lee, Jeong-Hoon Kim, Razia Sultana, Sun Yeou Kim, Ui Joung Youn, Jin-Hyoung Kim

**Affiliations:** 1 Division of Polar Life Sciences, Korea Polar Research Institute, Incheon 21990, Korea; seulah@kopri.re.kr (S.L.); hdw@kopri.re.kr (D.-W.H.); ickim@kopri.re.kr (I.-C.K.); jhkim94@kopri.re.kr (J.-H.K.); 2 Seoul School of Integrated Sciences & Technologies (aSSIST), Seoul 03767, Korea; 3 Research Unit of Cryogenic Novel Material, Korea Polar Research Institute, Incheon 21990, Korea; mhkoo1016@kopri.re.kr (M.H.K.); junhyucklee@kopri.re.kr (J.H.L.); 4 Polar Science, University of Science & Technology, Incheon 21990, Korea; 5 Department of Pharmacy, Jagannath University, Dhaka 1100, Bangladesh; raziasultana.du@gmail.com; 6 College of Pharmacy, Gachon University, 191 Hambakmoero, Yeonsu-gu, Incheon 21936, Korea; sunnykim@gachon.ac.kr

**Keywords:** Antarctic fish, *Notothenia rossii*, *Champsocephalus gunnari*, omega-3, polyunsaturated fatty acids, MMP-1 inhibition

## Abstract

Total fatty-acid (FA) contents of different organs (stomach, liver, brain, and skin) of two Antarctic fish, marbled rockcod (*Notothenia rossii*) and mackerel icefish (*Champsocephalus gunnari*), were examined using gas chromatography–mass spectrometry (GC–MS). *N. rossii* possessed higher contents of total omega-3, where eicosapentaenoic acid (EPA) and docosahexaenoic acid (DHA), the most represented omega-3 FAs, were distributed throughout all parts of the fish. The highest level of EPA was observed in the skin and that of DHA was observed in the brain of *N. rossii*. *C. gunnari* showed organ peculiarity in that most of the omega-3 FAs were found in stomach and skin. Specifically, the highest levels of EPA and DHA were both observed in the stomach. Although *N. rossii* and *C. gunnari* both inhabit the Antarctic Southern Oceans, their characteristics in terms of the composition of fatty acids were shown to vary. The extracts were also evaluated for matrix metalloproteinase-1 (MMP-1)-inhibitory activities in UVB-induced human dermal fibroblasts, where extracts of the skin and liver of *N. rossii* showed the most significant inhibition upon MMP-1 production. These findings provide experimental evidence that the extracts of the Antarctic fish could be utilized as bioactive nutrients, particularly in the enhancement of skin health.

## 1. Introduction

Skin is the largest external barrier in human body that protects against physical, chemical, and biological insults [1,2]. The skin is composed of the epidermis and dermis, where collagen is the major component of the dermis and is associated with other molecules to stabilize the organization of the extracellular matrix. Skin aging (photoaging) is a complex biological process involving histological and morphological changes caused by intrinsic (e.g., genetic and endogenous) and extrinsic (e.g., UV rays and contaminants) factors [1,2]. Progression of photoaging causes skin fragility, laxity, blister formation, and formation of wrinkles [3]. UV radiation (more specifically UVB) induces photoaging via penetrating the epidermis, as well as the upper layer of the dermis. It increases the production of various matrix metalloproteinases (MMPs), which ultimately accelerates the progressive collagen degradation [3,4,5]. Among the MMPs, matrix metalloproteinase-1 (MMP-1), also known as human fibroblast collagenase, is responsible for most of collagen breakdown in skin, which initiates the cleavage of type I, III, VII, VIII, and X collagen [3,6]. In this regard, omega-3 polyunsaturated fatty acids (PUFAs) are attracting attention as potential agents for maintenance of skin health, as well as treatment of skin disorders, especially those mediated by UV radiation [7], and fish oil containing omega-3 PUFAs (docosahexaenoic acid (DHA) and eicosapentaenoic acid (EPA)) has previously been reported with UVB-protective effects through several studies [8,9].

Notothenioids, belonging to the order Perciformes, are mainly distributed throughout the Southern Ocean around the coasts of New Zealand, southern South America, and Antarctica [10], and they account for more than 70% of Antarctic fish [11]. Among eight families that belong to the suborder Notothenioidei, the Nototheniidae and the Channichthyidae constitute the species considered as most valuable to international fisheries, with marbled rockcod (*Notothenia rossii*) and mackerel icefish (*Champsocephalus gunnari*) being the representatives of the respective families [12]. In addition to the value of fishery resources, these Antarctic fishes, which are the dominant species of the Antarctic Southern Oceans, have been studied and evaluated as potential biological indicators [13,14,15], and there are number of studies that examined the fatty-acid (FA) composition of notothenioid species [16,17,18]; however, only a few studies analyzed that of Antarctic fish in the South Georgia region [12]. According to a previous report that fish inhabiting cold water contain higher levels of PUFAs, mainly DHA and EPA, compared to those inhabiting the warm water [19], the two representative species of the Antarctic oceans, *N. rossii* and *C. gunnari*, were expected to possess high contents of PUFAs.

**Figure 1:**
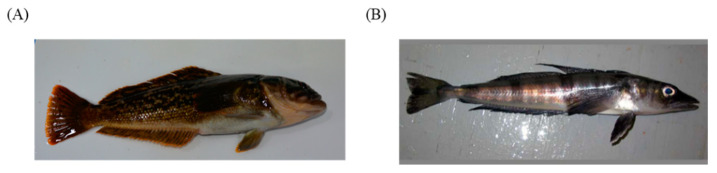
Experimental fish: (**A**) *Notothenia rossii*; (**B**) *Champsocephalus gunnari*.

In the current study, *N. rossii* and *C. gunnari* were chosen as representative commercial Antarctic species having a similar ecological distribution, where both species inhabit the Antarctic Southern Oceans (Figure 1). *N. rossii* is a demersal species and *C. gunnari* is a benthopelagic species, whereas the depth of water they usually live in is similar in the range of 5–350 m [20]. The two species collected from South Shetland Islands were compared with regard to their fatty-acid contents. This is the first study to report the omega-3, total FA, and cholesterol contents, along with MMP-1-inhibitory activity, of different organs of the Antarctic notothens.

## 2. Results and Discussion

### 2.1. GC–MS Analysis of the Fish Extracts

The two Antarctic fish *N. rossii* and *C. gunnari* were dissected into four parts, stomach, liver, brain, and skin, and the dried organs were extracted with hexane at room temperature. The extracts were subjected to methylation to obtain fatty-acid methyl esters (FAMEs) and were analyzed by gas chromatography–mass spectrometry (GC–MS). The GC–MS chromatograms and the summary of GC–MS data are shown in Figure S1 and Table S1.

#### 2.1.1. Brain

**Figure 2:**
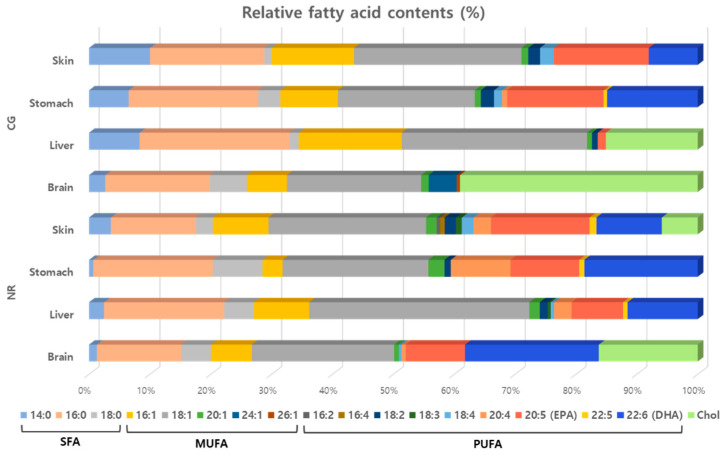
Bar representations of relative content (%) of fatty acids of different organs of *N. rossii* and *C. gunnari*.

The relative FA and cholesterol contents of *N. rossii* and *C. gunnari* are shown in Table 1 and Figure 2. The contents of saturated FAs (SFAs) and monounsaturated FAs (MUFAs) were slightly higher in *C. gunnari* (Table 1), with the most predominant SFA and MUFA being palmitic acid (16:0) and oleic acid (18:1), respectively. It was notable that *C. gunnari* contained rare long-chain MUFAs, (*Z*)-15-tetracosenoic acid (24:1) and (*Z*)-19-hexacosenoic acid (26:1). Among various MUFA, the contents of total omega-9 and omega-7 were 24.15% and 6.71% in *N. rossii* and 26.22% and 9.63% in *C. gunnari*, with oleic acid and palmitoleic acid being the most predominant omega-9 and omega-7 FAs, respectively. The most remarkable difference between the two species was that no polyunsaturated FAs (PUFAs) were detected in *C. gunnari*, while the most prominent omega-3 FAs, eicosapentaenoic acid (EPA, 20:5) and docosahexaenoic acid (DHA, 22:6), were found in *N. rossii* with relative contents of 9.75% and 21.98%, respectively. Another type of omega-3, stearidonic acid (18:4), and arachidonic acid (AA, 20:4), an omega-6 FA, were also detected in *N. rossii* in small quantity. According to previous reports estimating the lipid composition of fish brain to be affected by dietary input [21,22], the distinct difference in the FA profiles of the two species might be due to their different feeding habit, where *N. rossii* is known to feed mainly on zooplankton, zoobenthos, and small fish [23], and *C. gunnari* is known to feed on krill and other fish [24]. High levels of cholesterol were observed in both species, where that of *C. gunnari* was more than twofold higher (16.25% in *N. rossii* and 39.04% in *C. gunnari*). It was previously reported that many factors influence cholesterol content in fish, and PUFA content is inversely related to cholesterol content [25]. This supported the patterns of the PUFA and cholesterol profile of *C. gunnari*; however, it was not clearly applicable in the case of *N. rossii*.

#### 2.1.2. Liver

The relative content of SFA was higher in *C. gunnari* (34.48%) compared to *N. rossii* (27.10%), and that of MUFA was similar in both species (Table 1). The contents of omega-9 FAs (predominantly oleic acid) were higher in *N. rossii* (37.22%) than in *C. gunnari* (29.05%), while that of omega-7 FAs (palmitoleic acid) was twofold higher in *C. gunnari* (16.90%) compared to *N. rossii* (8.43%). Significant differences were again observed in the contents of PUFAs, where only trace amounts of 7,10-octadecadienoic acid (18:2) and EPA were detected in *C. gunnari*, while various other types of omega-3 FAs (7,10-octadecadienoic acid (18:2), linolenic acid (18:3), stearidonic acid (18:4), and docosapentaenoic acid (22:5)) along with omega-6 FAs (arachidonic acid, AA (20:4)) were detected in *N. rossii*, along with EPA (8.45%) and DHA (11.54%) (Table S2). Another difference was observed in the cholesterol contents, where 15.07% was detected in *C. gunnari*, while no cholesterol was detected in the case of *N. rossii*. This result is consistent with a previous report that PUFA content is inversely related to cholesterol content [25]. According to a previous study, FAs in the liver are generally subjected to oxidation or esterification to produce glycerol or cholesterol, for further synthesis of triglycerides and cholesterol esters [26]. This may explain the low PUFA content in *C. gunnari*, as opposed to the high cholesterol content.

#### 2.1.3. Stomach

The relative contents of SFAs and MUFAs were slightly higher in *C. gunnari* (SFAs: 28.51% in *N. rossii* and 31.44% in *C. gunnari*, MUFAs: 29.89% in *N. rossii* and 32.94% in *C. gunnari*), as shown in Table 1. The level of omega-9 FAs (predominantly oleic acid) was higher in *N. rossii* (26.09%) compared to *C. gunnari* (23.48%), and that of omega-7 FAs (palmitoleic acid) was 8.38% in *C. gunnari*, while a lower level was detected in *N. rossii* (3.30%). In contrast, the contents of PUFAs were higher in *N. rossii* (41.61% in *N. rossii* and 35.62% in *C. gunnari*). The levels of omega-3 FAs were similar in both fish; however, a significant difference in the contents of omega-6 PUFAs (AA, 20:4) was observed, which serves as a primary eicosanoid precursor. Furthermore, 9.79% AA was detected in *N. rossii*, whereas only 0.81% AA was detected in *C. gunnari*. In both species, cholesterol content was 0%, and high levels of EPA (11.28% in *N. rossii* and 15.85% in *C. gunnari*) and DHA (18.63% in *N. rossii* and 14.89% in *C. gunnari*) were observed. Other types of PUFA detected included 7,10-octadecadienoic acid (18:2) and docosapentaenoic acid (22:5), while stearidonic acid (18:4) was only detected in *C. gunnari*. The abovementioned differences between the two species might be due to different diet preferences [26], since the fish were captured at different sites of the Admiralty Bay.

#### 2.1.4. Skin

The difference in the FA profiles of *N. rossii* and *C. gunnari* was noticeable, where SFA contents were 20.35% and 29.96%, and MUFA contents were 36.76% and 42.21%, respectively (Table 1). The content of omega-9 FAs (predominantly oleic acid) was similar in both species (28.63% in *C. gunnari* and 27.62% in *N. rossii*), while that of omega-7 FAs (palmitoleic acid) was higher in *C. gunnari* (13.58%) compared to *N. rossii* (7.65%). As in other organs of the fish, the total PUFA content was higher in *N. rossii* (36.98%) compared to *C. gunnari* (27.83%). Levels of EPA and DHA were slightly higher in *N. rossii* (16.19% EPA and 10.74% DHA) than in *C. gunnari* (15.62% EPA and 8.04% DHA), and different kinds of other PUFAs were detected in *N. rossii* (9,12-hexadecadienoic acid (16:2), 6,9,12,15-hexadecatetraenoic acid (16:4), 7,10-octadecadienoic acid (18:2), linolenic acid (18:3), stearidonic acid (18:4), AA (20:4), and docosapentaenoic acid (22:5)). No cholesterol was observed in *C. gunnari*, whereas 5.89% was detected in *N. rossii*. The relatively high level of PUFAs in the skin of both species can be explained by previous reports that fish skins generally constitute high PUFA content and are a prime material for producing PUFA [27,28].

### 2.2. Cell Viability

**Figure 3:**
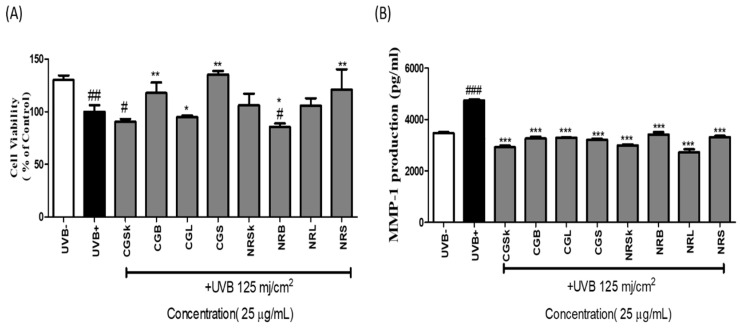
(**A**) Cell viability and (**B**) inhibitory activity upon UVB-induced MMP-1 production of extracts of different organs of *C. gunnari* (CG) and *N. rossii* (NR). SK = skin, B = brain, L = liver, S = stomach. Each value is represented as the mean ± SEM of triplicate experiments. (*) *p* < 0.05, (**) *p* < 0.01, and (***) *p* < 0.001 compared to UVB treatment group; (#) *p* < 0.05, (##) *p* < 0.01, and (###) *p* < 0.001 compared to normal control group.

Extracts of different organs of *N. rossii* and *C. gunnari* were evaluated for the viability of NHDF cells. The obtained results from the cell viability assay indicated that the UVB-irradiated group was found with the lowest cell viability as compared to the normal control group. According to our data, upon treatment with skin, brain, liver, and stomach extracts of both *N. rossii* and *C. gunnari*, there was no noticeable cell death in the UVB-damaged condition at 25 µg/mL as compared to the UVB-induced group or the normal group (Figure 3A). However, the skin extract of *C. gunnari* and brain extract of *N. rossii* were noted with the lowest cell viability compared to other extracts.

### 2.3. MMP-1-Inhibitory Activity

Extracts of different organs of *N. rossii* and *C. gunnari* were evaluated for MMP-1-inhibitory activity in UVB-induced conditions (125 mJ/cm^2^). In comparison with the UVB-treated group, all extracts demonstrated potential inhibitory activity against MMP-1 in the UVB-induced condition, as shown in Figure 3B. According to the obtained results, extracts of *N. rossii* skin and liver demonstrated significant inhibitory activity against MMP-1 compared to both the UVB-irradiated group and the normal group, among other extracts of *N. rossii*. The skin extract of *C. gunnari* also exhibited high MMP-1-inhibitory effects although it was noted with cytotoxicity in comparison with the UVB treatment group (Figure 3B).

## 3. Discussion

The total FA and cholesterol composition of *N. rossii* and *C. gunnari* was described in terms of four different organs: brain, liver, stomach, and skin. The contents of SFAs and MUFAs were somewhat higher in all parts of *C. gunnari* compared to those of *N. rossii*. As can be expected from the higher MUFA level, omega-7 FAs, which are among the most prominent MUFAs, were detected in all parts of *C. gunnari* with higher quantity, while omega-9 FAs, which are also predominant MUFAs, were found to be higher in liver and stomach of *N. rossii*. The highest level of omega-7 FAs was observed in liver of *C. gunnari*, while the liver of *N. rossii* contained the highest level of omega-9 FAs. Palmitoleic acid, a representative omega-7 FA, is known to have antidiabetic effects by enhancing insulin production and secretion, increasing fat breakdown, and reducing fat synthesis and storage [29]. Its anti-inflammation properties have also been highlighted in previous studies, where it reduced inflammation in LPS-stimulated macrophages by inhibiting NF-κB [29,30]. Recently, it was also reported that topical treatment of palmitoleic acid resulted in wound healing via anti-inflammatory effects [31]. Oleic acid, a predominant omega-9 FA that is rich in olive oil, is characterized by various beneficial effects on human health. It is reported to inhibit the proliferation of breast cancer cells [32,33,34], as well as prevent colorectal cancer development [35,36]. In one study, oleic acid was shown to suppress the overexpression of a well-known oncogene, HER2 (erbB-2), which plays a key role in the progression of several human cancers [37]. Furthermore, oleic acid was reported to serve as an anti-inflammatory agent [38] and to be responsible for the hypotensive activity of olive oil [39]. On the other hand, the contents of total PUFAs were higher in *N. rossii* throughout all organs, and each species had the highest level of total PUFAs in the stomach. Omega-3 FAs were distributed in all four organs of *N. rossii*, with the highest level of EPA being observed in the skin and that of DHA being observed in the brain. In the case of *C. gunnari*, the highest levels of EPA and DHA were both observed in the stomach. Unlike *N. rossii*, *C. gunnari* exhibited organ peculiarity in that no PUFAs were detected in the brain, and only a low level of PUFAs was detected in the liver. Most of the PUFAs were detected in the stomach and skin of *C. gunnari*, a great proportion being omega-3 FAs. By comparing the total FA contents of *N. rossii* and *C. gunnari*, it was observable that *N. rossii* possessed approximately twofold higher relative omega-3 content in total. EPA and DHA are well known for a vast array of health benefits. They are known to be essential for proper fetal development and healthy aging [40], and DHA is reported to play a crucial role in the development of the fetal brain and retina [41]. EPA and DHA are also known to have anti-inflammatory and antioxidative effects [42]. Their anti-inflammatory properties are associated with improvement of cardiovascular functions, which alters lipid metabolism, induces hemodynamic changes, decreases arrhythmias, improves endothelial function, and modulates platelet function [43,44]. EPA and DHA have also been studied in regard to Alzheimer’s disease (AD), and they were shown to improve cognitive functioning in mild AD patients [45]. According to previous reports that fish oil containing omega-3 PUFAs exhibits UVB-protective effects [8,9], the extracts of different organs of *N. rossii* and *C. gunnari* were evaluated for their MMP-1-inhibitory activity, where all extracts showed inhibition against UVB-induced NHDF cells, with extracts of *N. rossii* skin and liver exhibiting the most significant inhibitory activity against MMP-1 compared to both the UVB-irradiated group and the normal group.

## 4. Materials and Methods

### 4.1. Animals

Adult marbled rockcod, *Notothenia rossii* (*n* = 5), was collected by line and hook at the depth of 30–50 m in Admiralty Bay (King George Island, Antarctica Sejong Island) in January 2019, and the frozen mackerel icefish, *Champsocephalus gunnari* (*n* = 5), was provided by a deep-sea fishing company (Jeong Il) in May 2020. Both species were collected from the same area of South Shetland Islands. In general, securing Antarctic fish is difficult in terms of timing and accessibility, and there were limitations to obtain species other than these two. The frozen fish were dissolved at room temperature, then dissected and washed in tertiary distilled water, and freeze-dried. The FA composition was examined in the stomach, liver, brain, and skin.

### 4.2. Chemicals and Reagents

DMEM (Dulbecco’s modified Eagle’s medium), FBS (fetal bovine serum), penicillin, and streptomycin were obtained from Gibco-BRL (Grand Island, NY, USA). ELISA kits (Human Total MMP-1) were from R&D Systems Inc. (Minneapolis, MN, USA).

### 4.3. Lipid Extraction

Each frozen organ (stomach, liver, brain, and skin) was weighed. After crushing each of the dried organs finely, they were transferred into a 100 mL bottle. Next, 20–50 mL of hexane was poured, before shaking several times and extracting for half a day at room temperature. After concentrating the extracts using a concentrator, 50 mg of each sample was added to a round flask. Then, 1 mL of 14% BF_3_-methanol was added, before the flask was closed with a stopper, bathed for 5 min at 80 °C using a thermostat, and cooled. A saturated aqueous NaCl solution (3 mL) and hexane (3 mL) were added to each sample, followed by shaking. Finally, aliquots of the upper layers with fatty-acid methyl esters (FAMEs) were obtained and dried with anhydrous Na_2_SO_4_.

### 4.4. Fatty-Acid Analyses

Routine GC analyses were performed on a TRACE 1300 series GC equipped with the TSQ series (MS) and Triplus RSH autosampler (Thermo Fisher Scientific, Waltham, MA, USA), with a DB-WAX (30 m × 0.32 mm id × 0.25 μm) column at an initial temperature of 50 °C for 1 min followed by a 5 °C/min increase until reaching 170 °C, maintained for 5 min, and then 2 °C/min increase until reaching 220 °C, maintained for 10 min. Helium was used as the carrier gas, flowing at 1.0 mL/min, with an inlet temperature of 220 °C. The sample peaks were identified by the retention times obtained through comparison with NIST 2014 MS Library. The relative amount of each FA was calculated by integrating the area under the peak and dividing the result by the total area for all FAs.

### 4.5. Cell Culture

Normal human dermal fibroblasts (NHDFs; cat. no. PCS-201-012™) were purchased from the American Type Culture Collection (ATCC) and cultured in high-glucose DMEM (Thermo Scientific, Waltham, MA, USA) with 10% FBS and 1% penicillin–streptomycin (PS). Cultured cells were maintained in a humidifier at 37 °C and 5% CO_2_. NHDF cells used for this study were between passage numbers five and 10. Pretreatment of UVB irradiation was performed following a previously reported method with slight modification [46] implementing UVB (125 mJ/cm^2^) using a UVB machine (Bio-Link BLX-312; Vilber Lourmat GmbH, Marne-la-Vallée, France).

### 4.6. Cell Viability Assay

The MTT assay was performed to check the cell viability. NHDF (3 × 10^3^ cells/well) cells were seeded into 48-well plates, and cells were incubated further for 24 h at 37 °C in 5% CO_2_. UVB radiation was performed following previously described method with slight modification [46,47]. Briefly, NHDF cells were suspended in a small amount of PBS and then exposed to UVB (125 mJ/cm^2^) for around 40 s by using a UVB machine (Bio-Link BLX-312; Vilber Lourmat GmbH, Marne-la-Vallée, France) followed by washing with warm PBS three times. After washing with PBS, the NHDF cells were treated with extracts of the skin, brain, liver, and stomach of both *N. rossii* and *C. gunnari* (NRSK, NRB, NRL, NRS, CGSK, CGB, CGL, and CGS) and incubated for another 48 h. Then, the culture medium was removed, and 0.5 mg/mL of the MTT solution was added after 24 h and 48 h of incubation. After 1 h, the MTT solution was removed, and 100 µL of DMSO was added to each well. Then, the absorbance was estimated at a wavelength of 570 nm using a microplate reader (Molecular Devices E09090; San Francisco, CA, USA).

### 4.7. Measurement of MMP-1 in NHDF Cells

NHDF (3 × 10^3^ cells/well) cells were seeded into 48-well plates for 24 h of incubation at 37 °C in 5% CO_2_. Then, the cells were treated with extracts of skin, brain, liver, and stomach of both *N. rossii* and *C. gunnari* (NRSK, NRB, NRL, NRS, CGSK, CGB, CGL, and CGS) (25 µg/mL) in the presence or absence of UVB irradiation (125 mJ/cm^2^) separately, following the previously described method. After 48 h of incubation, the concentrations of MMP-1 were analyzed from the preserved supernatant using commercially available ELISA kits (Human Total MMP-1; R&D Systems Inc.) following the manufacturers’ instructions. Each sample was analyzed in triplicate.

### 4.8. Statistical Analysis

The results were presented as the mean ± standard error of the mean from three independent experiments. GraphPad Prism 5 (GrahPad Software Inc., La Jolla, CA, USA) was used for performing statistical analysis. One-way analysis of variance, followed by the Newman–Keuls multiple comparison tests, was performed, and *p* < 0.05 was considered as statistically significant.

## 5. Conclusions

In summary, the chemical profiles of extracts of different organs of the two Antarctic fish, *N. rossii* and *C. gunnari*, were examined to investigate their total fatty-acid (FA) contents, and the extracts were also evaluated for their inhibitory effects against matrix metalloproteinase-1 (MMP-1) production. All parts of *N. rossii* possessed high contents of total omega-3 FAs, while the stomach and skin possessed most of the omega-3 FAs in the case of *C. gunnari*. Omega-7 FAs, representative monounsaturated FAs (MUFAs), were higher in *C. gunnari* in all parts, while omega-9 FAs, also important MUFAs, were higher in the liver and stomach of *N. rossii*, and in the brain and skin of *C. gunnari*. All extracts showed inhibitory activity upon UVB-induced MMP-1 production; among them, the extracts of the skin and liver of *N. rossii* showed potential antiaging effects by exhibiting the most significant inhibition. 

Although *N. rossii* and *C. gunnari* have similar ecological distributions, where both inhabit the Antarctic oceans within the range of temperature of −1.9 °C to 1.5 °C, the fatty-acid composition of each species varied, particularly when different organs of the fish were compared. Despite different characteristics in the composition of PUFAs, the two Antarctic fish are considered to possess high contents of PUFAs, and further research needs to be carried out to compare the fatty-acid contents of the Antarctic fish and fish from temperate regions. The current study suggests a possible approach for the polyunsaturated FA (PUFA)-rich Antarctic fish to be viewed as a potential bioactive nutrients or even therapeutic agents, for enhancing skin health and preventing/treating skin photoaging, which can be further investigated to reveal its molecular mechanism along with in vivo study.

## Figures and Tables

**Table 1:** Total FA and cholesterol contents (%) of different organs of *N. rossii* and *C. gunnari*.

Species	Part	SFA	MUFA	PUFA		Cholesterol
				Omega-3	Other PUFA	
NR	Brain	20.04	30.86	32.21	0.64	16.25
	Liver	27.10	47.02	21.81	4.16	0.00
	Stomach	28.51	29.89	30.76	10.85	0.00
	Skin	20.35	36.76	30.99	5.99	5.89
CG	Brain	25.95	35.01	0.00	0.00	39.04
	Liver	34.48	48.16	1.34	0.95	15.07
	Stomach	31.44	32.94	32.66	2.96	0.00
	Skin	29.96	42.21	25.90	1.93	0.00

NR = *N. rossii*, CG = *C. gunnari*.

## Data Availability

Not applicable.

## Supplementary Materials

The following supporting information can be downloaded at https://www.mdpi.com/article/10.3390/molecules27144554/s1: Figure S1. GC–MS chromatograms of extracts of different organs of *C. gunnari* (CG) and *N. rossii* (NR); Table S1. Summary of GC–MS data; Table S2: Detailed FA composition (%) of different organs of *N. rossii* and *C. gunnari*.

## Sample Availability

Samples of the compounds are available from the authors.

## References

[1] Wong Q.Y.A., Chew F.T. (2021). Defining Skin Aging and Its Risk Factors: A Systematic Review and Meta-Analysis. Sci. Rep..

[2] Kirkwood K.L. (2018). Inflammaging. Immunol. Investig..

[3] Moon H.J., Lee S.R., Shim S.N., Jeong S.H., Stonik V.A., Rasskazov V.A., Zvyagintseva T., Lee Y.H. (2008). Fucoidan Inhibits Uvb-Induced Mmp-1 Expression in Human Skin Fibroblasts. Biol. Pharm. Bull..

[4] Fisher G.J., Datta S.C., Talwar H.S., Wang Z.-Q., Varani J., Kang S., Voorhees J.J. (1996). Molecular Basis of Sun-Induced Premature Skin Ageing and Retinoid Antagonism. Nature.

[5] Kohl E., Steinbauer J., Landthaler M., Szeimies R.M. (2011). Skin Ageing. J. Eur. Acad. Dermatol. Venereol..

[6] Dong K.K., Damaghi N., Picart S.D., Markova N.G., Obayashi K., Okano Y., Masaki H., Grether-Beck S., Krutmann J., Smiles K.A. (2008). Uv-Induced DNA Damage Initiates Release of Mmp-1 in Human Skin. Exp. Dermatol..

[7] Pilkington S.M., Watson R.E.B., Nicolaou A., Rhodes L.E. (2011). Omega-3 Polyunsaturated Fatty Acids: Photoprotective Macronutrients. Exp. Dermatol..

[8] Huang T.-H., Wang P.-W., Yang S.-C., Chou W.-L., Fang J.-Y. (2018). Cosmetic and Therapeutic Applications of Fish Oil’s Fatty Acids on the Skin. Mar. Drugs.

[9] Engström K., Saldeen A.-S., Yang B., Mehta J.L., Saldeen T. (2009). Effect of Fish Oils Containing Different Amounts of Epa, Dha, and Antioxidants on Plasma and Brain Fatty Acids and Brain Nitric Oxide Synthase Activity in Rats. Upsala J. Med. Sci..

[10] Eastman J., Grande L. (1989). Evolution of the Antarctic Fish Fauna with Emphasis on the Recent Notothenioids. Geol. Soc. Lond. Spec. Publ..

[11] Eastman J.T. (2005). The Nature of the Diversity of Antarctic Fishes. Polar Biol..

[12] Stowasser G., Pond D.W., Collins M.A. (2012). Fatty Acid Trophic Markers Elucidate Resource Partitioning within the Demersal Fish Community of South Georgia and Shag Rocks (Southern Ocean). Mar. Biol..

[13] Machado C., Zaleski T., Rodrigues E., dos Santos Carvalho C., Cadena S.M.S.C., Gozzi G.J., Krebsbach P., Rios F.S.A., Donatti L. (2014). Effect of Temperature Acclimation on the Liver Antioxidant Defence System of the Antarctic Nototheniids Notothenia Coriiceps and Notothenia Rossii. Comp. Biochem. Physiol. Part B Biochem. Mol. Biol..

[14] Rodrigues E., Feijó-Oliveira M., Vani G., Suda C., Carvalho C., Donatti L., Lavrado H. (2013). Interaction of Warm Acclimation, Low Salinity, and Trophic Fluoride on Plasmatic Constituents of the Antarctic Fish Notothenia Rossii Richardson, 1844. Fish Physiol. Biochem..

[15] De Souza M.R.D.P., Herrerias T., Zaleski T., Forgati M., Kandalski P.K., Machado C., Silva D.T., Piechnik C.A., Moura M.O., Donatti L. (2018). Heat Stress in the Heart and Muscle of the Antarctic Fishes Notothenia Rossii and Notothenia Coriiceps: Carbohydrate Metabolism and Antioxidant Defence. Biochimie.

[16] Lund E., Sidell B. (1992). Neutral Lipid Compositions of Antarctic Fish Tissues May Reflect Use of Fatty Acyl Substrates by Catabolic Systems. Mar. Biol..

[17] Sidell B.D., Crockett E.L., Driedzic W.R. (1995). Antarctic Fish Tissues Preferentially Catabolize Monoenoic Fatty Acids. J. Exp. Zool..

[18] Hagen W., Kattner G., Friedrich C. (2000). The Lipid Compositions of High-Antarctic Notothenioid Fish Species with Different Life Strategies. Polar Biol..

[19] Benitez L.V., De Silva S.S. (1989). Amino Acid and Fatty Acid Profiles in Aquaculture Nutrition Studies. Fish Nutrtiton Research in Asia: Proceedings of the Third Asian Fish Nutrition Network Meeting.

[20] Miller R.G. (1993). History and Atlas of the Fishes of the Antarctic Ocean.

[21] Pagliarani A., Pirini M., Trigari G., Ventrella V. (1986). Effect of Diets Containing Different Oils on Brain Fatty Acid Composition in Sea Bass (*Dicentrarchus labrax* L.). Comp. Biochem. Physiol. B Comp. Biochem..

[22] Sharma P., Kumar V., Sinha A.K., Ranjan J., Kithsiri H., Venkateshwarlu G. (2010). Comparative Fatty Acid Profiles of Wild and Farmed Tropical Freshwater Fish Rohu (*Labeo rohita*). Fish Physiol. Biochem..

[23] Stepanowska K., Nędzarek A. (2020). Changes in the Body Chemical Composition and the Excretion of Nitrogen and Phosphorus During Long-Term Starvation of Antarctic Fish Notothenia Coriiceps and Notothenia Rossii. Eur. Zool. J..

[24] Weber K., Goerke H. (1996). Organochlorine Compounds in Fish Off the Antarctic Peninsula. Chemosphere.

[25] Osman H., Suriah A., Law E. (2001). Fatty Acid Composition and Cholesterol Content of Selected Marine Fish in Malaysian Waters. Food Chem..

[26] Magalhães B., Fiamoncini J., Deschamps F., Curi R., Silva L. (2010). Comparison of Fatty Acid Composition in Nine Organs of the Sympatric Antarctic Teleost Fish Species Notothenia Coriiceps and Notothenia Rossii (Perciformes: Nototheniidae). Comp. Biochem. Physiol. Part B Biochem. Mol. Biol..

[27] Sahena F., Zaidul I., Jinap S., Jahurul M., Khatib A., Norulaini N. (2010). Extraction of Fish Oil from the Skin of Indian Mackerel Using Supercritical Fluids. J. Food Eng..

[28] Zuta C.P., Simpson B.K., Chan H.M., Phillips L. (2003). Concentrating Pufa from Mackerel Processing Waste. J. Am. Oil Chem. Soc..

[29] Morse N. (2015). Lipid-Lowering and Anti-Inflammatory Effects of Palmitoleic Acid: Evidence from Preclinical and Epidemiological Studies. Lipid Technol..

[30] Souza C.O., Teixeira A.A., Biondo L.A., Silveira L.S., Calder P.C., Rosa Neto J.C. (2017). Palmitoleic Acid Reduces the Inflammation in Lps-Stimulated Macrophages by Inhibition of NFΚB, Independently of PPARs. Clin. Exp. Pharmacol. Physiol..

[31] Weimann E., Silva M.B.B., Murata G.M., Bortolon J.R., Dermargos A., Curi R., Hatanaka E. (2018). Topical Anti-Inflammatory Activity of Palmitoleic Acid Improves Wound Healing. PLoS ONE.

[32] Chajès V., Thiébaut A.C., Rotival M., Gauthier E., Maillard V., Boutron-Ruault M.-C., Joulin V., Lenoir G.M., Clavel-Chapelon F. (2008). Association between Serum Trans-Monounsaturated Fatty Acids and Breast Cancer Risk in the E3n-Epic Study. Am. J. Epidemiol..

[33] Escrich E., Solanas M., Moral R., Grau L., Costa I., Vela E., Escrich R. (2008). Dietary Lipids and Breast Cancer: Scientific Clinical, Anatomopathological and Molecular Evidences. Rev. Esp. De Obes..

[34] Martin-Moreno J.M., Willett W.C., Gorgojo L., Banegas J.R., Rodriguez-Artalejo F., Fernandez-Rodriguez J.C., Maisonneuve P., Boyle P. (1994). Dietary Fat, Olive Oil Intake and Breast Cancer Risk. Int. J. Cancer.

[35] Macquart-Moulin G., Riboli E., Cornée J., Charnay B., Berthezene P., Day N. (1986). Case-Control Study on Colorectal Cancer and Diet in Marseilles. Int. J. Cancer.

[36] Stoneham M., Goldacre M., Seagroatt V., Gill L. (2000). Olive Oil, Diet and Colorectal Cancer: An Ecological Study and a Hypothesis. J. Epidemiol. Community Health.

[37] Carrillo Pérez C., Cavia Camarero M.d.M., Alonso de la Torre S. (2012). Antitumor Effect of Oleic Acid; Mechanisms of Action. A Review. Nutr. Hosp..

[38] Carrillo Pérez C., Cavia Camarero M.d.M., Alonso de la Torre S. (2012). Role of Oleic Acid in Immune System; Mechanism of Action; a Review. Nutr. Hosp..

[39] Teres S., Barceló-Coblijn G., Benet M., Alvarez R., Bressani R., Halver J.E., Escriba P. (2008). Oleic Acid Content Is Responsible for the Reduction in Blood Pressure Induced by Olive Oil. Proc. Natl. Acad. Sci. USA.

[40] Dunstan J.A., Mitoulas L.R., Dixon G., Doherty D.A., Hartmann P.E., Simmer K., Prescott S.L. (2007). The Effects of Fish Oil Supplementation in Pregnancy on Breast Milk Fatty Acid Composition over the Course of Lactation: A Randomized Controlled Trial. Pediatric Res..

[41] Ramakrishnan U., Stein A.D., Parra-Cabrera S., Wang M., Imhoff-Kunsch B., Juárez-Márquez S., Rivera J., Martorell R. (2010). Effects of Docosahexaenoic Acid Supplementation During Pregnancy on Gestational Age and Size at Birth: Randomized, Double-Blind, Placebo-Controlled Trial in Mexico. Food Nutr. Bull..

[42] Bloomer R., Larson D., Galpin A., Fisher-Wellman K., Schilling B. (2009). Effect of Eicosapentaenoic and Docosahexaenoic Acid on Resting and Exercise-Induced Inflammation and Oxidative Stress. J. Int. Soc. Sports Nutr..

[43] Ebrahimi M., Ghayour-Mobarhan M., Rezaiean S., Hoseini M., Parizade S.M.R., Farhoudi F., Hosseininezhad S.J., Tavallaei S., Vejdani A., Azimi-Nezhad M. (2009). Omega-3 Fatty Acid Supplements Improve the Cardiovascular Risk Profile of Subjects with Metabolic Syndrome, Including Markers of Inflammation and Auto-Immunity. Acta Cardiol..

[44] Cottin S., Sanders T., Hall W. (2011). The Differential Effects of Epa and Dha on Cardiovascular Risk Factors. Proc. Nutr. Soc..

[45] Swanson D., Block R., Mousa S.A. (2012). Omega-3 Fatty Acids Epa and Dha: Health Benefits Throughout Life. Adv. Nutr..

[46] Subedi L., Lee T.H., Wahedi H.M., Baek S.-H., Kim S.Y. (2017). Resveratrol-Enriched Rice Attenuates Uvb-Ros-Induced Skin Aging Via Downregulation of Inflammatory Cascades. Oxidative Med. Cell. Longev..

[47] Hwang E., Kim S.H., Lee S., Lee C.H., Do S.G., Kim J., Kim S.Y. (2013). A comparative study of baby immature and adult shoots of Aloe vera on UVB-induced skin photoaging in vitro. Phyther. Res..

