# Supplementary material for: Comparison of Fatty Acid Contents and MMP-1 Inhibitory Effects of the Two Antarctic Fish, Notothenia rossii and Champsocephalus gunnari
Source: Molecules. 2022 Jul 17;27(14):4554. doi: 10.3390/molecules27144554 (PMC9317339; doi:10.3390/molecules27144554)
Supplement: Supplementary file 1 [file molecules-27-04554-s001.zip › molecules-1765763-supplementary.pdf]

Supplementary Material

Comparison of Fatty Acid Contents and MMP-1 Inhibitory Effects of the Two Antarctic Fish, *Notothenia rossii* and *Champsocephalus gunnari*

Seulah Lee [1,2], Man Hyung Koo [3], Dong-Won Han [1], Il-Chan Kim [1], Jun Hyuck Lee [3,4], Jeong-Hoon Kim [1], Razia Sultana [5], Sun Yeou Kim [6], Ui Joung Youn [1,4,*] and Jin-Hyoung Kim [1,4,*]

[1] Division of Polar Life Sciences, Korea Polar Research Institute, Incheon 21990, Korea

[2] Seoul School of Integrated Sciences & Technologies (aSSIST), Seoul 03767, Korea

[3] Research Unit of Cryogenic Novel Material, Korea Polar Research Institute, Incheon 21990, Korea

[4] Polar Science, University of Science & Technology, Incheon 21990, Korea

[5] Department of Pharmacy, Jagannath University, Dhaka-1100, Bangladesh

[6] College of Pharmacy, Gachon University, 191 Hambakmoero, Yeonsu-gu, Incheon, 21936, Korea

* Co-correspondence:

Ui Joung Youn, E-mail: ujyoun@kopri.re.kr

Jin-Hyoung Kim, E-mail: kimjh@kopri.re.kr

List of Electronic Supplementary Material

Figure S1. GC-MS chromatograms of extracts of different organs of *C. gunnari* (CG) and *N. rossii* (NR)

Table S1. Summary of GC-MS data

Table S2. Detailed FA composition (%) of different organs of *N. rossii* and *C. gunnari*

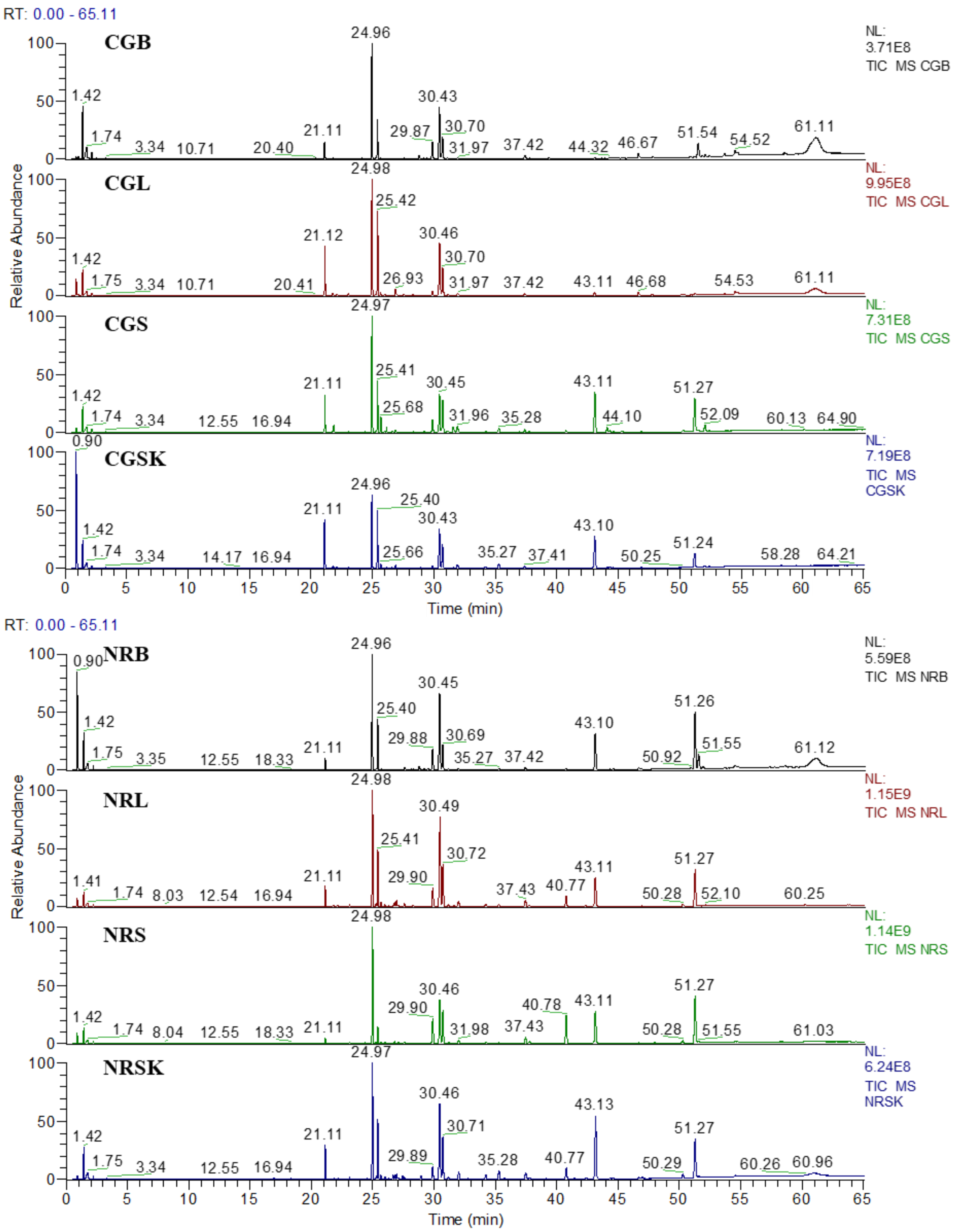

Figure S1. GC-MS chromatograms of extracts of different organs of *C. gunnari* (CG) and *N. rossii* (NR); B=brain, L=liver, S=stomach, SK=skin

Table S1. Summary of GC-MS data

Retention Time (min)	Observed Fatty Acid Methyl Esters (*m/z*)	Observed Fragmentation Patterns (*m/z*)	Elemental Composition	Identification
21.1	242.23	74, 87, 143, 199	$C_{14}H_{28}O_2$	Tetradecanoic acid
24.9	270.24	74, 87, 143, 227	$C_{16}H_{32}O_2$	Palmitic acid
25.4	268.25	55, 69, 74, 83, 96, 110, 152, 194, 236	$C_{16}H_{30}O_2$	9-Hexadecenoic acid
29.8	298.30	74, 87, 143, 199, 255, 298	$C_{18}H_{36}O_2$	Stearic acid
30.4	296.26	55, 69, 83, 97, 98, 114, 180, 222, 264	$C_{18}H_{34}O_2$	Oleic acid
31.9	294.31	67, 81, 95, 96, 97, 123, 150, 222, 264	$C_{18}H_{32}O_2$	7,10-Octadecadienoic acid
35.2	-	79, 91, 93, 105, 119, 133, 161, 175, 189, 221	$C_{18}H_{28}O_2$	Stearidonic acid (6,8,12,15-Octadecatetraenoic acid)
37.4	324.34	55, 69, 83, 97, 98, 111, 123, 208, 250, 292	$C_{20}H_{38}O_2$	11-Eicosenoic acid
40.7	-	79, 91, 93, 105, 119, 133, 150, 175, 203, 217, 247	$C_{20}H_{32}O_2$	Arachidonic acid
43.1	-	79, 91, 93, 119, 133, 147, 161, 201, 215, 247	$C_{20}H_{30}O_2$	5,8,11,14,17-Eicosapentaenoic acid
50.2	-	79, 91, 93, 105, 119, 131, 145, 175, 208, 225, 274	$C_{22}H_{34}O_2$	7,10,13,16,19-Docosapentaenoic acid
51.2	-	79, 91, 105, 119, 131, 145, 159, 173, 241	$C_{22}H_{32}O_2$	4,7,10,13,16,19-Docosahexaenoic acid
54.5	-	55, 69, 83, 97, 98, 111, 133, 151, 180, 222, 264	$C_{18}H_{34}O_2$	(*Z*)11-Octadecenoic acid

61.1	386.37	105, 145, 213, 275, 301, 368	$C_{27}H_{46}O$	Cholesterol

Table S2. Detailed FA composition (%) of different organs of *N. rossii* and *C. gunnari*

										**PUFA**									
		**SFA**			**MUFA**								**n-3**		**n-6**	**n-3**			
		**14:0**	**16:0**	**18:0**	**16:1**	**18:1**	**20:1**	**24:1**	**26:1**	**16:2**	**16:4**	**18:2**	**18:3**	**18:4**	**20:4**	**20:5 (EPA)**	**22:5**	**22:6 (DHA)**	**Chol**
**NR**	**Brain**	1.31	13.8	4.93	6.71	23.38	0.77							0.48	0.64	9.75		21.98	16.25
	**Liver**	2.48	19.74	4.88	9.11	36.19	1.72					1.29	0.5	0.58	2.87	8.45	0.74	11.54	
	**Stomach**	0.71	19.63	8.17	3.3	23.95	2.64					1.06			9.79	11.28	0.85	18.63	
	**Skin**	3.6	13.96	2.79	9.14	25.88	1.74			0.57	0.74	1.85	0.97	1.96	2.83	16.19	1.13	10.74	5.89
**CG**	**Brain**	2.67	17.15	6.13	6.55	22.07	1.25	4.58	0.56										39.04
	**Liver**	8.32	24.61	1.55	16.9	30.46	0.8					0.95				1.34			15.07
	**Stomach**	6.54	21.25	3.65	9.46	22.47	1.01					2.15		1.36	0.81	15.85	0.56	14.89	
	**Skin**	10.04	18.75	1.17	13.58	27.5	1.13					1.93		2.24		15.62		8.04	

NR = *N. rossii*; CG = *C. gunnari*; Chol = cholesterol
